# VEGF188 promotes corneal reinnervation after injury

**DOI:** 10.1172/jci.insight.130979

**Published:** 2019-11-01

**Authors:** James T. Brash, Laura Denti, Christiana Ruhrberg, Franziska Bucher

**Affiliations:** 1 UCL Institute of Ophthalmology, London, United Kingdom.; 2 Department of Ophthalmology, University Hospital of Cologne, Cologne, Germany.

**Keywords:** Ophthalmology, Cardiovascular disease, Neurodegeneration, growth factors

## Abstract

Vascular endothelial growth factor A (VEGF) induces angiogenesis and vascular hyperpermeability in ocular tissues and is therefore a key therapeutic target for eye conditions in which these processes are dysregulated. In contrast, the therapeutic potential of VEGF’s neurotrophic roles in the eye has remained unexploited. In particular, it is not known whether modulating levels of any of the 3 major alternatively spliced VEGF isoforms might provide a therapeutic approach to promote neural health in the eye without inducing vascular pathology. Here, we have used a variety of mouse models to demonstrate differences in overall VEGF levels and VEGF isoform ratios across tissues in the healthy eye. We further show that VEGF isoform expression was differentially regulated in retinal versus corneal disease models. Among the 3 major isoforms — termed VEGF120, VEGF164, and VEGF188 — VEGF188 was upregulated to the greatest extent in injured cornea, where it was both necessary and sufficient for corneal nerve regeneration. Moreover, topical VEGF188 application further promoted corneal nerve regeneration without inducing pathological neovascularization. VEGF isoform modulation should therefore be explored further for its potential in promoting neural health in the eye.

## Abstract 2

The alternative VEGF splice form VEGF188 promotes nerve regeneration in corneal injury and may provide a therapeutic to treat corneal blindness.

## Graphical abstract

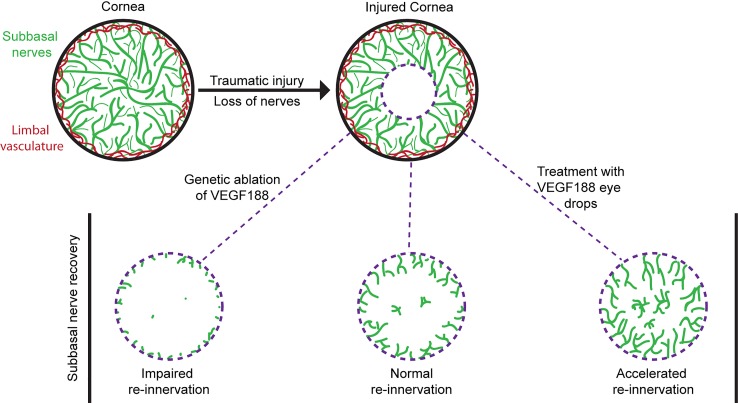

## Introduction

Therapies that target vascular endothelial growth factor A (referred to herein as VEGF) have been approved to treat several ocular conditions characterized by pathological new blood vessel growth, termed “ocular neovascularization” (1). Accordingly, antibody-based VEGF blockade is now the principal treatment for choroidal neovascularization in age-related macular degeneration (AMD) and macular edema in diabetic retinopathy (1). Anti-VEGF therapy is also used off-label to treat pathological corneal neovascularization (2, 3), which can develop due to inflammation, hypoxia, trauma, limbal stem cell deficiency, or corneal transplantation (4).

Despite these advances in treatment, several preclinical studies suggest that long-term anti-VEGF treatment may have adverse side effects, primarily because VEGF also has nonvascular functions (5). For example, treatment with VEGF-targeting antibodies reduces the survival of retinal ganglion cells (RGCs) in the mouse retina (6). Moreover, VEGF has neurotrophic functions for the corneal nerves that promote corneal health (7–9). Accordingly, topical anti-VEGF drugs, while decreasing pathological neovascularization in preclinical studies, also impair corneal epithelial repair and reinnervation following corneal injury (10). Moreover, several clinical studies reported side effects after topical anti-VEGF treatment that included persistent corneal epithelial defects, as well as corneal perforation and melting, especially in patients with preexisting epithelial defects or neurotrophic keratopathy (11–13). Intravitreal anti-VEGF drugs, when used to treat a range of neovascular conditions such as neovascular AMD or macular edema, also decreased corneal nerve density and increased dry eye symptoms (14).

Before the approval of the currently prevailing antibody-based anti-VEGF therapies, an aptamer targeting one of several alternatively spliced VEGF isoforms was shown to improve vision in neovascular AMD (1), suggesting that targeting selective VEGF isoforms has therapeutic potential. However, it has not yet been examined whether anti-VEGF treatments that spare selective VEGF isoforms might preserve neuronal health in the retina or could be used to promote corneal reinnervation without inducing pathological blood vessel growth. Investigating these possibilities is important, because it is already known that the VEGF isoforms have both overlapping and nonredundant vascular and neuronal functions during normal organ development (5, 15). Here, we examined whether the 3 major VEGF isoforms, termed VEGF120, VEGF164, and VEGF188, make distinct contributions to mouse models of ocular injury. Whereas all 3 isoforms were upregulated during retinal ischemia in proportions similar to those in healthy retina, VEGF188 was upregulated more than VEGF120 or VEGF164 in models of corneal injury with or without neovascularization. Endogenous VEGF188, but not VEGF120 or VEGF164, was also required for nerve regeneration after corneal injury. Moreover, VEGF188-containing eye drops accelerated corneal nerve regeneration without promoting corneal neovascularization. Accordingly, modulating the VEGF isoform balance may provide a novel therapeutic approach for neuroprotection in corneal injury.

## Results

### VEGF isoform expression across ocular tissues.

**Figure 1:**
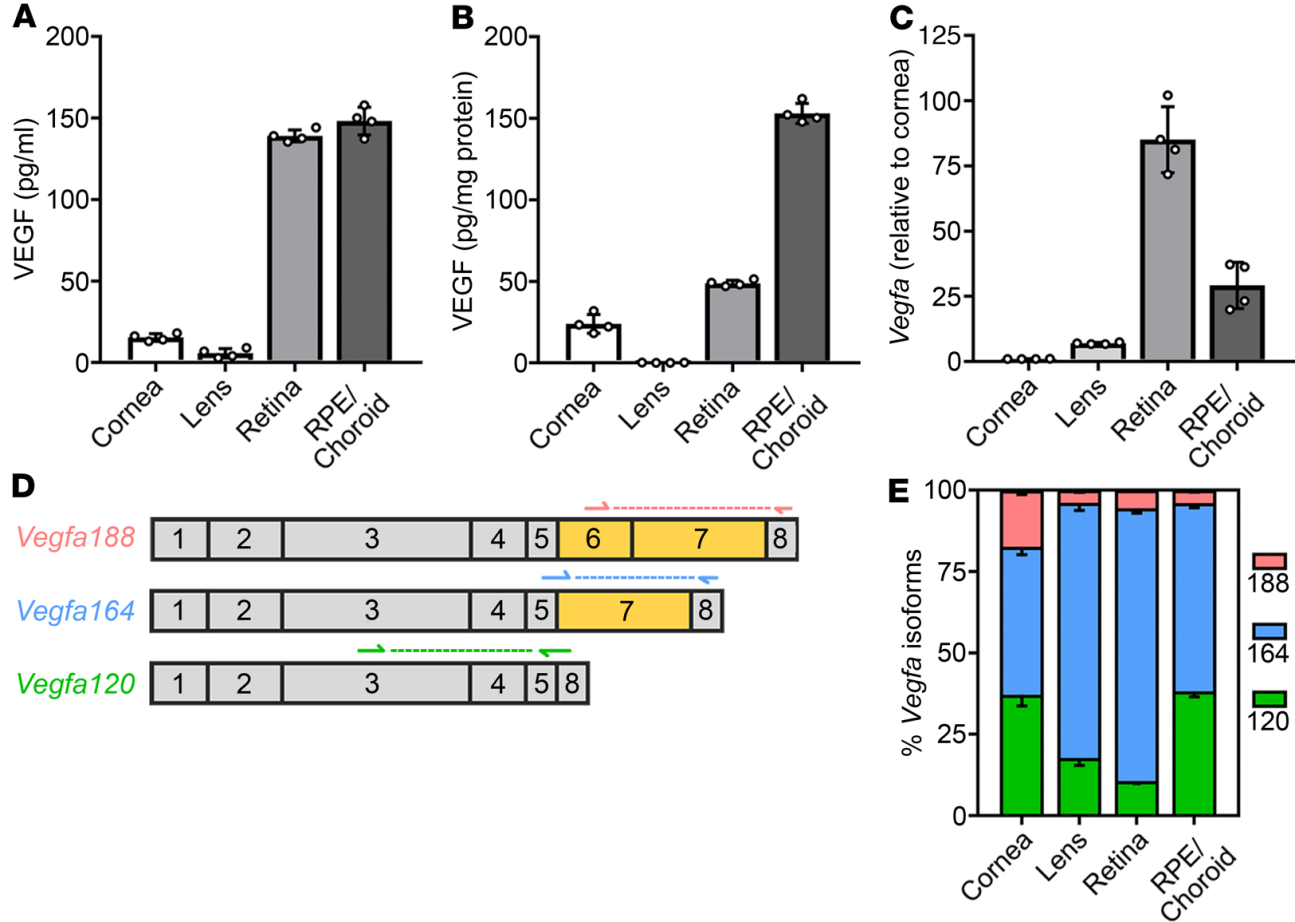
Comparison of VEGF expression across healthy adult ocular tissues. (**A** and **B**) VEGF protein content in ocular tissues from wild-type CD1 mice, determined by ELISA; *n* = 4 mice each; data are shown as mean ± SD per milliliter lysate (**A**) or as mean ± SD proportion of total protein in the lysates (**B**). (**C–E**) *Vegfa* transcript levels in ocular tissues from wild-type CD1 mice, determined by qRT-PCR; *n* = 4 mice each. (**C**) Total *Vegfa* expression for each tissue relative to expression in the cornea (mean ± SD). (**D**) Schematic representation of the exon structure of the 3 major mouse *Vegfa* isoform transcripts, including the alternatively spliced domains that encode the extracellular matrix–binding domains (yellow); the positions of the isoform-selective oligonucleotide primers used are indicated arrows, and the amplified region by dashed lines. (**E**) Proportions of *Vegfa* isoforms (mean; only the negative SD arm is shown). Each data point represents the value for pooled tissues from both eyes of 1 mouse (**A** and **B**) or the tissue from 1 eye of 1 mouse (**C**).

Building on prior work on ocular VEGF isoform expression (16–18), we compared VEGF protein and transcript levels in the cornea, lens, retina, and retinal pigment epithelium (RPE)/choroid complex of adult wild-type mice. In agreement with VEGF’s importance for RGC and choriocapillaris health (6, 19), VEGF protein was abundant in the retina and RPE/choroid (Figure 1, A and B, and Supplemental Figure 1, A and B; supplemental material available online with this article; https://doi.org/10.1172/jci.insight.130979DS1). In contrast, VEGF levels were low in the cornea and lens (Figure 1, A and B, and Supplemental Figure 1, A and B). This was expected, because lens and corneal avascularity is crucial for visual acuity, with some VEGF essential to provide neurotrophic support to corneal nerves (7–9). Quantitative reverse transcription PCR (qRT-PCR) corroborated that *Vegfa* expression was lowest in the lens and cornea and higher in the retina and RPE/choroid (Figure 1C). Interestingly, *Vegfa* expression in the RPE/choroid was almost 3-fold lower than in the retina, despite comparable VEGF protein levels in both sites. The difference in transcript and protein levels may indicate tissue-specific regulation of VEGF translation, turnover, or retention.

As reliable VEGF isoform antibodies are not presently available, we investigated the relative expression levels of the 3 major isoforms, VEGF120, VEGF164 and VEGF188, by qRT-PCR with *Vegfa* isoform–specific oligonucleotide primers (Figure 1D) (20). Consistent with prior work measuring *Vegfa* transcript abundance across the whole eye (18), *Vegfa164* was the most abundant and *Vegfa188* the least abundant isoform in each mouse eye tissue examined individually, including in the retina, lens, and RPE/choroid (16, 17), as well as in the cornea, which was not previously examined (Figure 1E). Notably, the isoform ratio differed according to tissue. Specifically, the relative amount of *Vegfa120* was higher in cornea and RPE/choroid compared with retina or lens, and the relative amount of *Vegfa188* was higher in the cornea than in any other ocular tissue examined.

### Retinal hypoxia increases the expression of all Vegfa isoforms.

**Figure 2:**
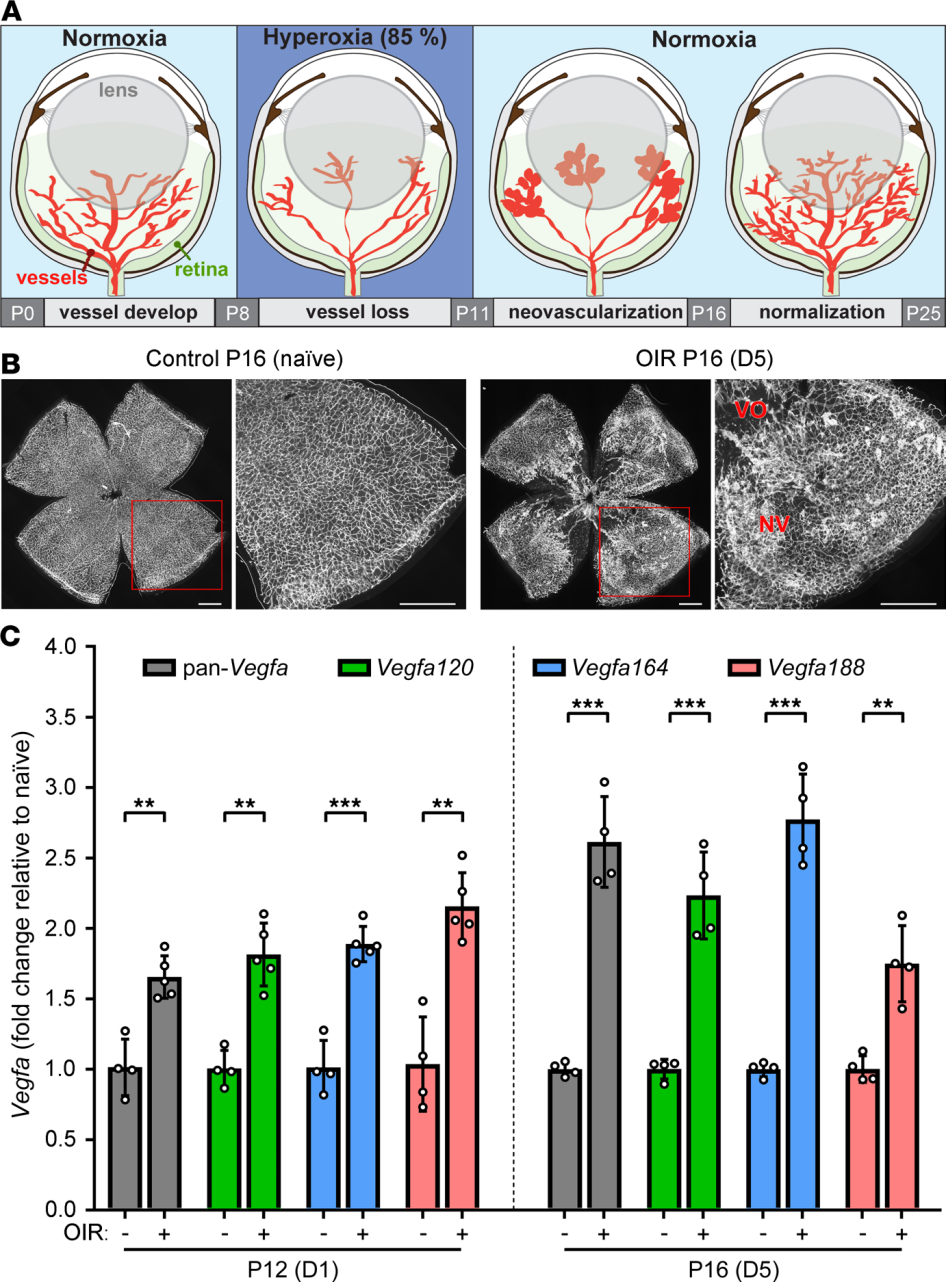
*Vegfa* isoform upregulation during OIR. (**A**) Schematic representation of the OIR protocol. Mouse pups are reared in 85% oxygen from P8 to P11 to induce retinal vaso-obliteration. After return to normoxia, VEGF-driven neovascularization occurs, peaking on P16 before onset of vascular normalization. (**B**) IB4-stained retinal whole mounts from littermate P16 wild-type mice housed in normoxia (naive) or subjected to OIR and analyzed on day 5 (D5) after return to normoxia. Red squares indicate areas shown at higher magnification. Note central vaso-obliteration (VO) and the presence of neovascular tufts (NV) in the OIR model. Scale bars: 500 μm. (**C**) Total *Vegfa* and *Vegfa* isoform expression in the retinas of P12 (D1) and P16 (D5) wild-type mice reared in normoxia (–) or hyperoxia (+). Data are shown as mean fold change ± SD relative to control normoxia; *n* = 4 mice per age; each data point represents the value for 1 retina from 1 mouse. **P ≤ 0.01, ***P ≤ 0.001; 1-way ANOVA with Šidák’s multiple-comparisons test.

To determine whether specific VEGF isoforms are differentially regulated in neovascular eye disease caused by tissue ischemia, we studied their expression in a mouse model of oxygen-induced retinopathy (OIR), which has the hallmarks of retinopathy of prematurity and proliferative diabetic retinopathy (Figure 2, A and B, and Supplemental Figure 2, A and B) (21, 22). In this model, the immature retinal vasculature of pups exposed to transient hyperoxia regresses and fails to sustain retinal oxygen consumption on return to normoxia, causing *Vegfa* upregulation and thereby both beneficial retinal revascularization and pathological neovascular tufts (22, 23). To induce OIR, we exposed wild-type pups to either 75% oxygen for 5 days (21) or 85% oxygen for 3 days (24). We analyzed *Vegfa* isoform expression in the retina on days 1 and 5 after return to normoxia, i.e., shortly after ischemia induction and during peak neovascularization (24), respectively (Figure 2, A and B). As expected, both OIR protocols significantly increased *Vegfa* expression (Figure 2C and Supplemental Figure 2C). Consistent with a hypoxia response element in the *Vegfa* promoter that enables a rapid hypoxia response (25), *Vegfa* expression was already increased on day 1 and persisted to day 5 (Figure 2C). Among the total *Vegfa* transcript pool, the 3 isoforms were similarly upregulated (Figure 2C and Supplemental Figure 2). These findings suggest that hypoxia promotes a general increase in retinal *Vegfa* expression without affecting the default *Vegfa* splicing pattern to alter the isoform balance.

### Vegfa188 is upregulated more than Vegfa120 or Vegfa164 after corneal injury.

**Figure 3:**
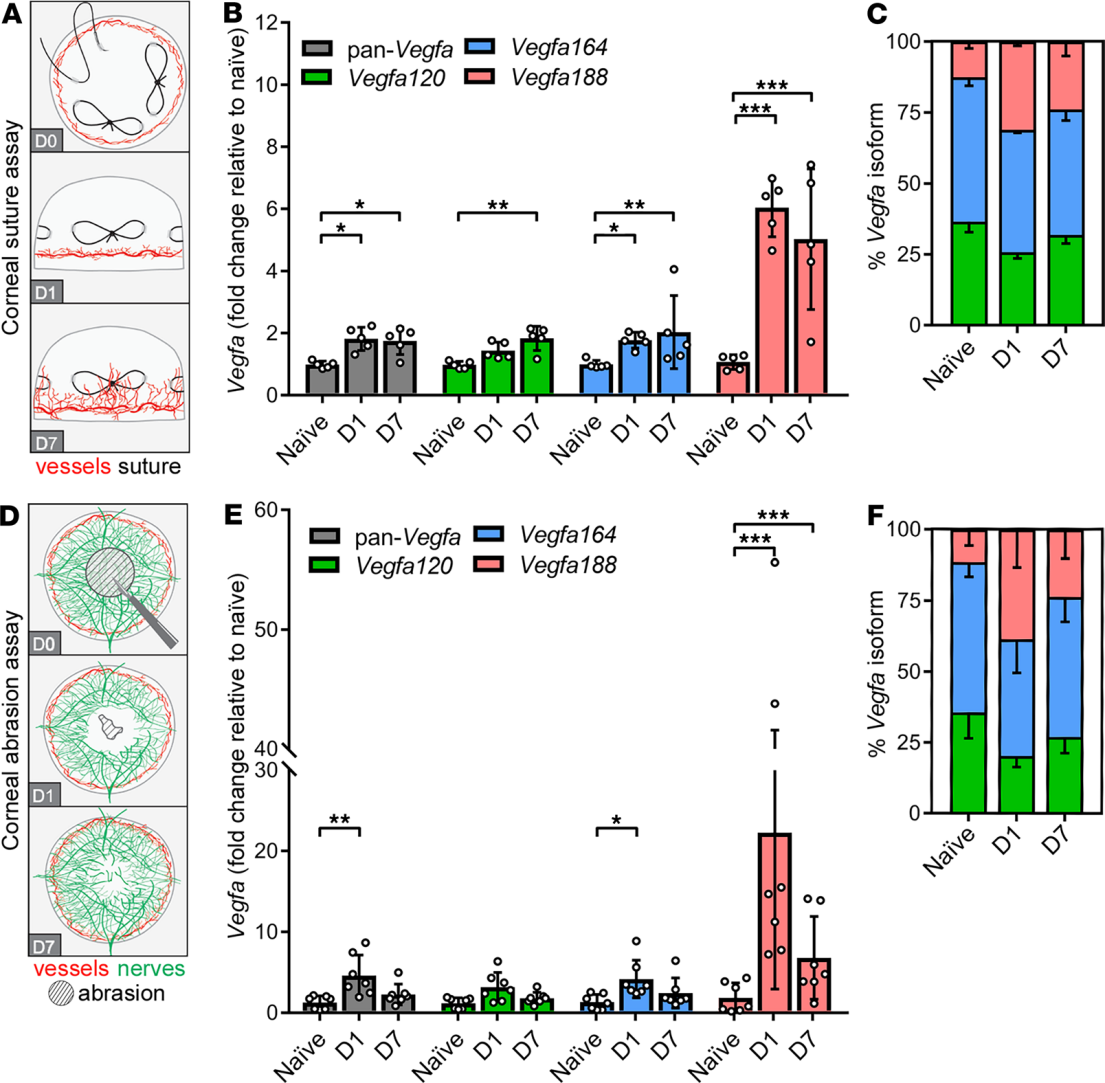
*Vegfa* isoform expression in models of corneal injury. (**A–C**) Corneal neovascularization. (**A**) Schematic representation of the corneal neovascularization assay in wild-type CD1 mice. Three sutures are placed into the corneal stroma (en face view; D0). Neovessels sprout from the limbus toward the sutures from D1 after surgery and reach the central cornea by D7 (lateral views). (**B** and **C**) Total *Vegfa* and *Vegfa* isoform expression, shown as (**B**) fold change in sutured versus naive corneas (mean ± SD) and (**C**) as proportions of isoforms (mean; only the negative arm of the SD is shown); *n* = 5 mice per condition; each data point represents the value for 1 cornea from 1 mouse. (**D–F**) Corneal abrasion. (**D**) Schematic representation of the corneal abrasion assay in wild-type CD1 mice (en face views). Central epithelium and subepithelial nerves are removed (D0). The epithelial wound is nearly closed on D1, but nerves have not yet regenerated. Reinnervation of the injured cornea is visible on D7. (**E** and **F**) Total *Vegfa* and *Vegfa* isoform expression, shown as (**E**) fold change in sutured versus naive corneas (mean ± SD) and (**F**) as proportions of isoforms (mean; only the negative arm of the SD is shown); *n* = 7 mice per condition; each data point represents the value for 1 cornea from 1 mouse. *P ≤ 0.05, **P ≤ 0.01, ***P ≤ 0.001; 1-way ANOVA with Šidák’s multiple-comparisons test.

Corneal VEGF levels increase in patients with corneal inflammation and in rodent models of corneal neovascularization (26, 27). Inflammation-induced neovascular outgrowth from the limbal vessels can be induced in mice by introducing sutures into the corneal stroma (Figure 3A) (28). When compared with naive cornea, *Vegfa* expression was significantly upregulated in the cornea on day 1 after suture placement, when inflammation had begun, and on day 7, when corneal neovascularization was advanced (Figure 3, A and B). Among the total *Vegfa* transcript pool, *Vegfa164* and *Vegfa188* were significantly upregulated at both time points, while *Vegfa120* expression was significantly increased only on day 7 (Figure 3B). Strikingly, *Vegfa188* transcripts were significantly more upregulated than either *Vegfa120* or *Vegfa164* transcripts on both days 1 and 7, therefore increasing the proportion of *Vegfa188* in the total *Vegfa* pool (Figure 3C and Supplemental Figure 3A). Accordingly, *Vegfa188* was the isoform upregulated to the greatest extent in mouse models of corneal neovascularization.

We next asked whether *Vegfa188* upregulation also occurs in a mouse model of corneal epithelial abrasion with subbasal nerve injury, which does not cause neovascularization (7). Whereas the epithelial wound healed rapidly between days 1 and 2 after injury in this model, the subbasal nerves regenerated gradually over the course of several weeks (Figure 3D and ref. 7). *Vegfa* expression was significantly upregulated on day 1 (Figure 3E). Among the total *Vegfa* transcript pool, both *Vegfa164* and *Vegfa188* were significantly upregulated on day 1, but *Vegfa188* upregulation was significantly higher than that of *Vegfa164* (Figure 3E and Supplemental Figure 3B). Total *Vegfa* transcript levels had declined again by day 7, but *Vegfa188* expression remained significantly upregulated (Figure 3E). Accordingly, *Vegfa188* comprised a greater proportion of the total *Vegfa* transcript pool after corneal abrasion (Figure 3F). Therefore, the *Vegfa* isoforms were differentially regulated in both models of corneal injury. The finding that VEGF188 upregulation persists into the prolonged period of corneal nerve regeneration may indicate a neurotrophic role for corneal nerves.

### Generation of mouse mutants to examine Vegfa isoform requirements in adult disease.

Although prior studies established VEGF’s role in corneal nerve regeneration (7–9), it is not known which VEGF isoforms are important. To compare the requirement of specific VEGF isoforms for corneal nerve regeneration, we generated *Vegfa* isoform mutants that selectively express VEGF120, VEGF164, or VEGF188. By combining a conditional null *Vegfa* allele (29) with a tamoxifen-inducible *Cre* transgene under the control of the ubiquitous chicken actin promoter (30), we circumvented the embryonic lethality of mice carrying even one loss-of-function allele of *Vegfa* (31, 32). Breeding the resulting *Vegfa^+/fl^ Cag-CreER^T2^* mice to mice with disrupted *Vegfa* splicing generated cryptic *Vegfa* isoform mutants that lack congenital neurovascular defects, but are amenable to postnatal VEGF isoform modulation. Thus, using the *Vegfa^120^* allele, which lacks *Vegfa164*- and *Vegfa188*-specific exons (33), we generated *Vegfa^120/fl^ Cag-CreER^T2^* offspring, in which tamoxifen injection inactivated the conditional *Vegfa*-null allele, so that the mice produced VEGF120, but not VEGF164 or VEGF188 (Supplemental Figure 4, A and B). Mice carrying the *Vegfa^164^* allele encoding VEGF164 only or the *Vegfa^188^* allele encoding VEGF188 only (34, 35) were used to generate analogous mutants expressing only VEGF164 or VEGF188, respectively (Supplemental Figure 4B). The resulting inducible *Vegfa* isoform mutants are useful for determining the postnatal requirements of specific VEGF isoforms in ocular health and other conditions.

### VEGF188 is essential and sufficient for corneal reinnervation following injury.

**Figure 4:**
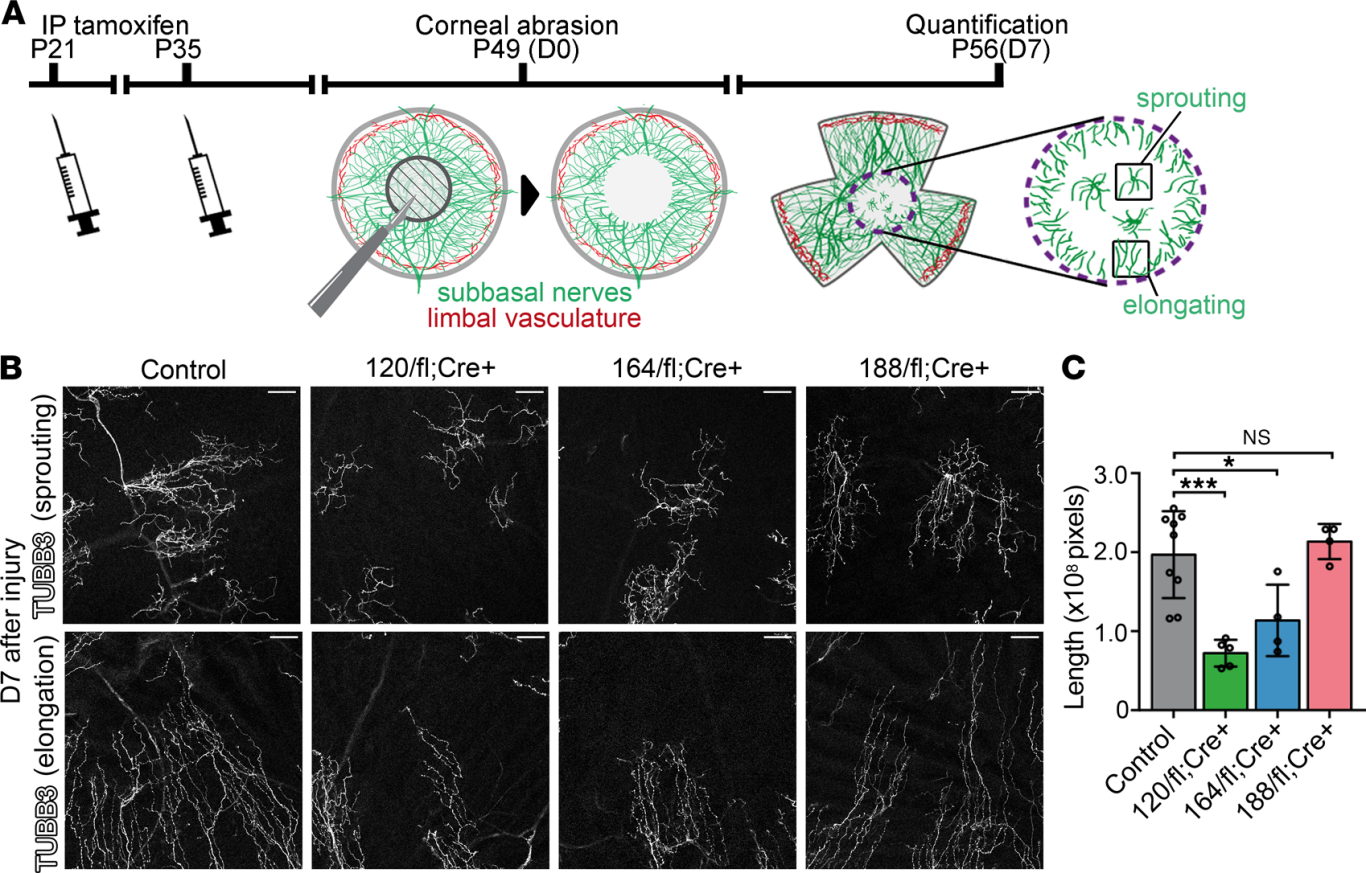
VEGF188 is essential and sufficient for reinnervation of injured cornea. (**A**) Experimental protocol of the corneal abrasion assay in mice expressing specific VEGF isoforms only (*Vegfa^xxx/fl^ Cag-CreER^T2^*). Mice of the indicated genotypes and pooled littermate controls (*Vegfa^+/+^*, *Vegfa^+/fl^*, *Vegfa^+/+^ Cag-CreER^T2^*) were injected intraperitoneally (IP) with tamoxifen on P21 and P35. Central corneal epithelial abrasion was performed on P49 and immunostaining of corneal flat mounts on P56; the dashed circle indicates the central area that was used for neurite quantification, with the 2 patterns of neurite regeneration illustrated in the higher-magnification image. (**B**) TUBB3 immunostaining of D7 corneal flat mounts to visualize subbasal plexus regeneration in tamoxifen-induced *Vegfa^fl/xxx^ Cag-CreER^T2^* mice (xxx/fl;Cre+) versus littermate controls. Scale bars: 50 μm. (**C**) Neurite length in the injured area of *Vegfa^xxx/fl^ Cag-CreER^T2^* mice (mean ± SD); *n* = 9 control mice, *n* = 5 120/fl;Cre+ mice, *n* = 4 164/fl;Cre+ mice, *n* = 4 188/fl;Cre+ mice; each data point represents the value for 1 cornea from 1 mouse. *P ≤ 0.05, ***P ≤ 0.001; 1-way ANOVA with Dunnett’s multiple-comparisons test.

To determine which specific VEGF isoform is essential for subbasal nerve regeneration, we performed corneal abrasion on tamoxifen-induced *Vegfa* isoform mutants (Figure 4A). In agreement with a prior report (7), control mice showed 2 patterns of nerve regeneration into the central area of injury: neurite sprouting from deeper, uninjured subepithelial nerves to form islands of centripetally extending neurites in the corneal center; and neurite elongation of subbasal nerves from outside the wound toward the corneal center (Figure 4, A and B). Both types of regeneration were also present in *Vegfa* isoform mutants, but only mice expressing VEGF188 had levels of neurite extensions similar to those of controls (Figure 4, B and C). Accordingly, VEGF188 was both necessary and sufficient for normal levels of corneal nerve regeneration.

### VEGF188 supplementation promotes corneal reinnervation following injury.

**Figure 5:**
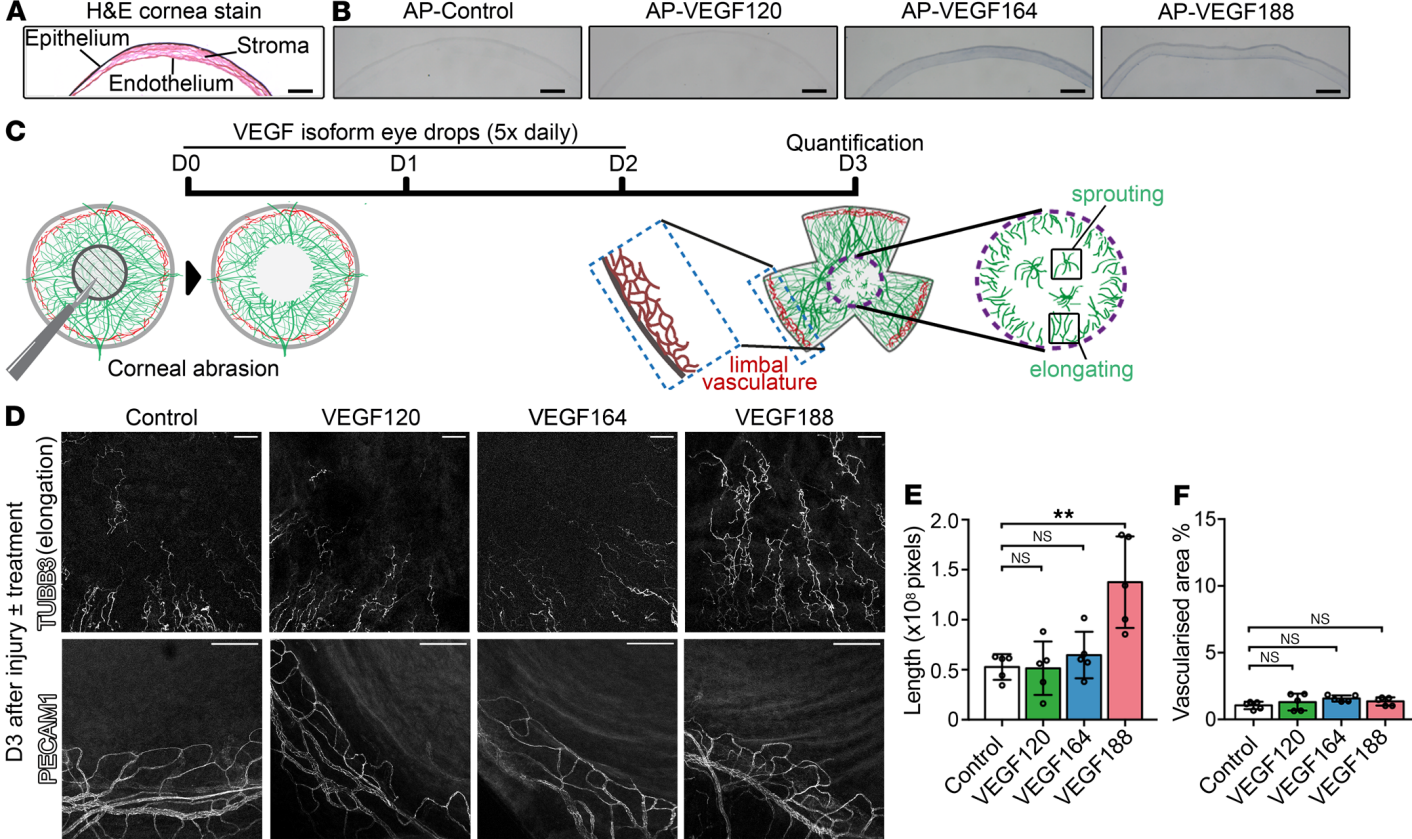
VEGF188 promotes reinnervation of the injured cornea. (**A**) H&E staining of corneal cross sections illustrates the layers of the wild-type adult mouse cornea. (**B**) VEGF isoform retention by corneal tissue was investigated in cross sections of fresh frozen, unfixed cornea from a wild-type CD1 mouse by incubation with AP protein (control) or the indicated AP-conjugated VEGF isoforms, followed by visualization of AP activity with substrate. Scale bars: 300 μm. *n* = 3 experiments. (**C**) Experimental protocol of the corneal abrasion assay in wild-type CD1 mice to compare the effects of VEGF isoforms on corneal nerve regeneration. Central corneal epithelial abrasion was performed on P49 CD1 mice (D0) and was followed by treatment with topical eye drops containing PBS (vehicle) or equal molar amounts of VEGF120, VEGF164, or VEGF188. Vehicle- or VEGF-containing eye drops were administered 5 times a day on D1, D2, and D3 after injury. Corneal flat mounts were immunostained on D3 after injury. (**D–F**) Immunostaining (**D**) of D3 corneal flat mounts for neuron-specific TUBB3 to visualize subbasal plexus regeneration (top row) and for PECAM-1 to visualize limbal blood vessels (bottom row) in wild-type CD1 mice after daily treatment from D1 to D3 with eye drops containing vehicle (control) or recombinant VEGF120, VEGF164, or VEGF188. Scale bars: 50 μm. (**E**) Neurite length (mean ± SD) in the central cornea and (**F**) vascular area in the corneal periphery (mean ± SD); *n* = 5 mice per treatment; each data point represents the value for 1 cornea from 1 mouse. **P ≤ 0.01; 1-way ANOVA with Dunnett’s multiple-comparisons test.

We next examined whether recombinant VEGF isoforms have the capacity to be retained by the cornea. Incubating fresh corneal tissue sections from wild-type mice with alkaline phosphatase–conjugated (AP-conjugated) recombinant VEGF isoforms and an AP substrate (20) showed that corneal tissue bound both VEGF188 and VEGF164, but not VEGF120 (Figure 5, A and B). We subsequently examined whether administering VEGF isoform–containing eye drops would accelerate neurite extension into the injured cornea (Figure 5C). As neurite extension was less prominent on day 3 than on day 7 (compare controls in Figure 4B and Figure 5D), we measured neurite extension on day 3 after injury (Figure 5, C and D). Consistent with poor binding to corneal tissue, VEGF120 did not promote neurite extension into the injured cornea compared with vehicle control (Figure 5, D and E). Even though VEGF164 was retained by corneal tissue (Figure 5B), it also did not promote neurite extension compared with control (Figure 5, D and E). By contrast, the VEGF188 isoform promoted significant neurite elongation into the injured cornea compared with control (Figure 5, D and E). The finding that exogenous VEGF188 accelerates neurite extension into the injured cornea suggests that endogenous VEGF188, despite its upregulation, remains rate limiting for corneal nerve regeneration. Remarkably, although corneal nerve regeneration was accelerated by topically applied VEGF188, there was no increase in blood vessel outgrowth from the limbus toward the injured cornea (Figure 5F). These findings raise the possibility that topical VEGF188 application may be an effective and safe approach to accelerate corneal nerve regeneration.

## Discussion

Here, we directly compared differences in *Vegfa* expression across healthy ocular tissues and in 3 models of eye disease. All adult ocular tissues contained *Vegfa* transcripts and VEGF protein, although VEGF protein was negligibly low in the lens. VEGF abundance in the retina and RPE/choroid was high, consistent with VEGF’s proposed trophic effects in these tissues beyond the period of vascularization during development. In contrast, VEGF abundance in the cornea was much lower, presumably to prevent vascular invasion from the corneal periphery while still maintaining local neurotrophic support (7–9). The ratio of *Vegfa* isoforms differed according to tissue, likely reflecting tissue-specific functions of the VEGF isoforms. For instance, the relative proportion of *Vegfa120* in the RPE/choroid *Vegfa* pool was greater than in the lens and retina *Vegfa* pools. VEGF120 is the most diffusible VEGF isoform, as it does not contain the extracellular matrix binding domains found in VEGF164 and VEGF188 (36). Accordingly, the RPE/choroid may express VEGF120 to support the choriocapillaris and RGCs from a distance (6, 16). Interestingly, the relative expression of *Vegfa188* was highest in the cornea, although a specific function for this isoform in the cornea had not previously been described.

VEGF is normally expressed at high levels in the fetal human retina (37) and the perinatal mouse retina (38, 39) to support blood vessel growth. We also observed, in agreement with prior work (23), upregulation of *Vegfa* transcripts in the OIR model of retinopathy of prematurity. Our finding that the 3 *Vegfa* isoforms were similarly upregulated suggests that retinal cell types do not modulate alternative VEGF splicing in response to hypoxia. Instead, the hypoxia response element in the *Vegfa* promoter (25) likely increases *Vegfa* transcription and is associated with the default pattern of splicing for this tissue, with high *Vegfa164* expression and substantially lower *Vegfa120* and *Vegfa188* expression.

Corroborating prior work on increased VEGF protein levels in corneal inflammation in patients (26) and a rat model of corneal neovascularization (27), we observed increased *Vegfa* transcription in 2 different mouse models of corneal injury, one causing inflammation-induced blood vessel growth and the other nerve damage–induced neurite sprouting. In both models, transcription of *Vegfa120* and *Vegfa164* was upregulated modestly, whereas *Vegfa188* showed striking upregulation compared with naive cornea. These findings raise the possibility that *Vegfa188* upregulation is a generic response in corneal injury that occurs independently of the primary pathological context, for example, chronic inflammation or nerve damage. The differences in alternative *Vegfa* splicing in injured cornea compared with retina may also relate to the different type of pathological insult, with hypoxia being the main cause of retinal ischemia. Moreover, the cornea and retina differ with respect to the cell types that are primary responders, i.e., epithelial cells in the cornea versus neural cells in the retina (40).

The expression of a soluble VEGFR1 decoy by corneal epithelium is thought to sequester excess VEGF in the cornea (41) and may be sufficient to prevent VEGF-induced neovascularization in the abrasion assay, in which corneal epithelium heals rapidly. In contrast, this protective mechanism appears insufficient to prevent neovascularization in the suture-induced model of corneal injury, which is characterized by a prolonged increase in VEGF due to both local transcription in corneal epithelium and the release of VEGF stores from recruited inflammatory cells (8). Although *Vegfa188* was the most upregulated isoform, mice expressing solely VEGF188 have less neovascularization in the corneal suture model than wild-type mice, as do mice expressing solely VEGF164 (42). Thus, it is likely that VEGF188 and VEGF164 cooperate with VEGF120 to promote corneal neovascularization (42), perhaps by forming a growth factor gradient that extends from the central injury site to the peripheral limbal vasculature, by analogy with *Vegfa* gradient formation during brain vascularization (35).

As prior studies had established the importance of VEGF for corneal nerve regeneration and because pan-VEGF blockade adversely affects corneal health, we investigated whether the 3 VEGF isoforms differentially regulate corneal nerve regeneration by generating and analyzing a complementary set of 3 VEGF isoform mouse mutant strains. These strains supersede in usefulness a prior set of *Vegfa* isoform mouse mutants, termed *Vegfa^120/120^*, *Vegfa^164/164^*, and *Vegfa^188/188^*, in which the constitutive expression of only one isoform causes congenital vascular defects (31, 34, 35, 43) that likely compound the pathological response of blood vessels to hypoxia and inflammation and therefore complicate attributing VEGF roles specifically to the injury response. Instead of inducing constitutive changes in the VEGF isoform composition that impact vascular development, we have generated cryptic *Vegfa* isoform mutants that develop and thrive with a full isoform set, but are amenable to isoform modulation at the time when a postnatal injury response is to be investigated. These *Vegfa* isoform mice should be useful for a range of investigations into the postnatal requirements of the individual VEGF isoforms in health and disease.

Here, we have used inducible *Vegfa* isoform mice to demonstrate that VEGF188 is both necessary and sufficient for corneal nerve regeneration. As exogenous delivery further accelerated nerve regeneration, VEGF188’s levels may remain rate limiting, despite a striking upregulation after injury. A likely explanation for VEGF188’s importance in corneal nerve regeneration might be its excellent retention in corneal tissue, which may be prolonged compared with that of VEGF164, because VEGF188 has 2 extracellular matrix–binding domains compared with only 1 for VEGF164 and none for VEGF120 (Figure 1D) (36). It remains unknown whether VEGF188 binds corneal neurites directly to promote reinnervation, or instead binds the residual corneal epithelium or preserved corneal stroma to promote the secretion of proneural factors. To resolve these possibilities, it would be essential to compare the corneal epithelium and corneal sensory nerves for their expression of the 4 VEGF receptors (VEGFR1, VEGFR2, NRP1, and NRP2) before selective removal of the relevant receptors in these tissues.

We conclude that adverse effects of global anti-VEGF therapy to treat corneal neovascularization may result from an inadvertent inhibition of VEGF188’s beneficial effects on corneal nerve regeneration. Conversely, VEGF188 supplementation may provide a novel therapeutic avenue to enhance endogenous VEGF188 effects for corneal nerve regeneration, perhaps in combination with other neurotrophic factors and antiinflammatories.

## Methods

### Animals.

All mice were bred at UCL, including wild-type CD1 and C57BL6/J mice, and the following genetically modified mouse strains: *Vegfa^120^*, *Vegfa^164^* and *Vegfa^188^* (33–35), *Vegfa^fl^* (29), and *Cag-CreER^T2^* (30), which were maintained on a mixed genetic background (C57BL6/J and 129/Sv).

### OIR.

C57BL6/J pups were housed in 85% oxygen with a CD1 foster mother from P8 to P11, after which pups were returned to normoxia to induce retinal ischemia. Pups were culled for RNA analysis or to confirm OIR by immunostaining. In some experiments, pups were housed in 75% oxygen from P7 to P12 and culled for RNA analysis or immunostaining. Littermate pups reared in normoxia served as experimental controls.

### Induction of corneal injury.

Mice were anesthetized by combined intraperitoneal injection of ketamine and dexmedetomidine (75 mg/kg and 0.5 mg/kg body weight, respectively). For local corneal anesthesia, 1 drop of oxybuprocaine (0.4%) was applied to the cornea after general anesthesia. Intraperitoneal atipamezole (1.0 mg/kg body weight) was used for reversal of general anesthesia. To induce corneal neovascularization, a 11-0 nylon suture (Serag Wiessner) was used to place 3 evenly spaced double loops into the corneal stroma of anesthetized mice; sutures were left in place for 1 or 7 days before mice were culled for RNA analysis or immunostaining. To induce corneal epithelial and nerve damage in anesthetized mice, a 1.5-mm trephine was used to mark the central cornea before a slit knife (Alcon) was used to erode a circular area of corneal epithelium and the corneal subbasal nerve plexus. Corneas were dissected from culled mice for RNA analysis on day 1 or 7 or for immunostaining on day 7. In some experiments, wild-type CD1 mice were treated from day 0 to day 2 with topical eye drops containing 52 μM VEGF120, VEGF164, or VEGF188 (ReliaTech GmbH) in 5 μL PBS or PBS only (vehicle control), and corneas were dissected and immunostained on day 3.

### VEGF ELISA.

Dissected ocular tissues were cut into small pieces and lysed in equal volumes of RIPA buffer (Merck) containing 0.1% SDS, protease inhibitor cocktail 2, and phosphatase inhibitor cocktail (Sigma-Aldrich). To aid lysis, the suspension was passed several times through a 23-G needle. The VEGF Quantikine ELISA kit (R&D Systems) was used to determine VEGF content, and the Bradford assay (Bio-Rad) was used to determine total protein concentration of the tissue lysates.

### Vegfa qRT-PCR.

RNA was extracted from tissues using TRI Reagent (Sigma-Aldrich) and cDNA synthesized using the Superscript IV reverse transcription kit (Thermo Fisher Scientific). For qRT-PCR analysis, cDNA was incubated with SYBR Green master mix (Applied Biosystems) containing gene-specific oligonucleotide primers and amplified on a QuantStudio 6 Flex Real-Time PCR system (Applied Biosystems). Fold change in gene expression was calculated using the ΔΔCt method (44), with expression of *Actb* used for normalization between samples. For statistical analysis, we compared ΔCt values to determine fold increase relative to control, or ΔΔCt values to determine differences in fold increase between isoforms. Absolute qRT-PCR was used to calculate the relative abundance of *Vegfa* isoforms in an individual sample, whereby qRT-PCR of serial dilutions of *Vegfa120*, *Vegfa164*, or *Vegfa188* cDNA was used to plot a standard curve of Ct value versus cDNA copy number. The following oligonucleotide primers were used for qRT-PCR: pan-*Vegfa* 5′-CAGATCATGCGGATCAAACCT-3′ and 5′-TTGTTCTGTCTTTCTTTGGTCTG-3′; *Vegfa120* 5′-GTAACGATGAAGCCCTGGAG-3′ and 5′-CCTTGGCTTGTCACATTTTTC-3’; *Vegfa164* 5′-CAGAACAAAGCCAGAAAATCAC-3′ and 5′-GCCTTGGCTTGTCACATCT-3′; *Vegfa188* 5′-AGTTCGAGGAAAGGGAAAGG-3′ and 5′-GCCTTGGCTTGTCACATCT-3′; *Actb* 5′-CACCACACCTTCTACAATGAG-3′ and 5′-GTCTCAAACATGATCTGGGTC-3′. Validation of the pan-*Vegfa* and *Vegfa* isoform–specific primers has been described (20, 35). Note that qRT-PCR detects some residual *Vegfa164* and *Vegfa188* transcript in tamoxifen-induced *Vegfa^164/fl^ Cag-CreER^T2^* and *Vegfa^188/fl^ Cag-CreER^T2^* mice, respectively (Supplemental Figure 4B). This is explained by incomplete nonsense-mediated decay of the mutant transcripts, which lack the essential exon 3 (45), because the isoform-specific primers are able to detect the nonsense transcript. While the knockdown of functional *Vegfa* transcripts is therefore underestimated for *Vegfa164* and *Vegfa188*, the primers used to evaluate levels of the *Vegfa120* transcripts do not detect nonsense transcript and therefore demonstrate the real level of knockdown.

### Whole-mount staining.

Retinas were dissected and fixed as described previously (46) and then whole-mount stained with rabbit anti–collagen IV (Bio-Rad, 2150-1470), Alexa Fluor 488–conjugated anti-rabbit IgG (Thermo Fisher Scientific, A11008) or biotin-conjugated isolectin B4 (IB4) (Sigma-Aldrich, L2140), and Alexa Fluor 647–conjugated streptavidin (Thermo Fisher Scientific, S21374), which were all diluted in PBS containing 10% serum-free protein block (Dako) and 0.1% Tween-20. Dissected corneas were fixed in 2% formaldehyde for 1 hour and then whole-mount stained with Cy3-conjugated neuron-specific beta-III tubulin (R&D Systems, NL1195R) or FITC-conjugated rat anti–mouse PECAM-1 (CD31) (BD Biosciences, 558738). Antibodies were diluted in PBS containing 3% BSA and 0.1% Triton X-100. Staining was visualized with an Eclipse Ti inverted epifluorescence microscope (Nikon).

### Statistics.

One-way ANOVA plus multiple-comparisons testing was used to determine significance; *P* values of 0.05 or less were considered significant.

### Study approval.

Animal procedures were performed in accordance with the Association for Research in Vision and Ophthalmology (ARVO) *Statement for the Use of Animals in Ophthalmic and Vision Research*, and approved by the Animal Welfare Ethical Review Body (AWERB; UCL Institute of Ophthalmology) and the UK Home Office.

## Author contributions

JTB, FB, and CR conceived and planned this study, analyzed data, and cowrote the manuscript. LD performed genetic crosses and genotyping. All authors reviewed and edited the manuscript.

## Supplementary Material

Supplemental data

## Version 1 11/01/2019

Electronic publication

## References

[1] Kim LA, D’Amore PA (2012). A brief history of anti-VEGF for the treatment of ocular angiogenesis. Am J Pathol.

[2] Keating AM, Jacobs DS (2011). Anti-VEGF treatment of corneal neovascularization. Ocul Surf.

[3] Stevenson W, Cheng SF, Dastjerdi MH, Ferrari G, Dana R (2012). Corneal neovascularization and the utility of topical VEGF inhibition: ranibizumab (Lucentis) vs bevacizumab (Avastin). Ocul Surf.

[4] Beebe DC (2008). Maintaining transparency: a review of the developmental physiology and pathophysiology of two avascular tissues. Semin Cell Dev Biol.

[5] Mackenzie F, Ruhrberg C (2012). Diverse roles for VEGF-A in the nervous system. Development.

[6] Nishijima K (2007). Vascular endothelial growth factor-A is a survival factor for retinal neurons and a critical neuroprotectant during the adaptive response to ischemic injury. Am J Pathol.

[7] Yu CQ, Zhang M, Matis KI, Kim C, Rosenblatt MI (2008). Vascular endothelial growth factor mediates corneal nerve repair. Invest Ophthalmol Vis Sci.

[8] Li Z, Burns AR, Han L, Rumbaut RE, Smith CW (2011). IL-17 and VEGF are necessary for efficient corneal nerve regeneration. Am J Pathol.

[9] Pan Z, Fukuoka S, Karagianni N, Guaiquil VH, Rosenblatt MI (2013). Vascular endothelial growth factor promotes anatomical and functional recovery of injured peripheral nerves in the avascular cornea. FASEB J.

[10] Kim EC, Lee WS, Kim MS (2010). The inhibitory effects of bevacizumab eye drops on NGF expression and corneal wound healing in rats. Invest Ophthalmol Vis Sci.

[11] Kim SW, Ha BJ, Kim EK, Tchah H, Kim TI (2008). The effect of topical bevacizumab on corneal neovascularization. Ophthalmology.

[12] Koenig Y, Bock F, Horn F, Kruse F, Straub K, Cursiefen C (2009). Short- and long-term safety profile and efficacy of topical bevacizumab (Avastin) eye drops against corneal neovascularization. Graefes Arch Clin Exp Ophthalmol.

[13] Galor A, Yoo SH Corneal melt while using topical bevacizumab eye drops. ophthalmic surg lasers imaging.

[14] Goldhardt R, Batawi HIM, Rosenblatt M, Lollett, Park JJ, Galor A (2019). Effect of anti-vascular endothelial growth factor therapy on corneal nerves. Cornea.

[15] Ruhrberg C (2003). Growing and shaping the vascular tree: multiple roles for VEGF. Bioessays.

[16] Saint-Geniez M, Maldonado AE, D’Amore PA (2006). VEGF expression and receptor activation in the choroid during development and in the adult. Invest Ophthalmol Vis Sci.

[17] Saint-Geniez M, Kurihara T, D’Amore PA (2009). Role of cell and matrix-bound VEGF isoforms in lens development. Invest Ophthalmol Vis Sci.

[18] Ng YS, Rohan R, Sunday ME, Demello DE, D’Amore PA (2001). Differential expression of VEGF isoforms in mouse during development and in the adult. Dev Dyn.

[19] Saint-Geniez M, Kurihara T, Sekiyama E, Maldonado AE, D’Amore PA (2009). An essential role for RPE-derived soluble VEGF in the maintenance of the choriocapillaris. Proc Natl Acad Sci USA.

[20] Tillo M (2015). VEGF189 binds NRP1 and is sufficient for VEGF/NRP1-dependent neuronal patterning in the developing brain. Development.

[21] Connor KM (2009). Quantification of oxygen-induced retinopathy in the mouse: a model of vessel loss, vessel regrowth and pathological angiogenesis. Nat Protoc.

[22] Smith LE (1994). Oxygen-induced retinopathy in the mouse. Invest Ophthalmol Vis Sci.

[23] Pierce EA, Avery RL, Foley ED, Aiello LP, Smith LE (1995). Vascular endothelial growth factor/vascular permeability factor expression in a mouse model of retinal neovascularization. Proc Natl Acad Sci USA.

[24] Villacampa P (2017). Accelerated oxygen-induced retinopathy is a reliable model of ischemia-induced retinal neovascularization. PLoS One.

[25] Forsythe JA (1996). Activation of vascular endothelial growth factor gene transcription by hypoxia-inducible factor 1. Mol Cell Biol.

[26] Philipp W, Speicher L, Humpel C (2000). Expression of vascular endothelial growth factor and its receptors in inflamed and vascularized human corneas. Invest Ophthalmol Vis Sci.

[27] Amano S, Rohan R, Kuroki M, Tolentino M, Adamis AP (1998). Requirement for vascular endothelial growth factor in wound- and inflammation-related corneal neovascularization. Invest Ophthalmol Vis Sci.

[28] Streilein JW, Bradley D, Sano Y, Sonoda Y (1996). Immunosuppressive properties of tissues obtained from eyes with experimentally manipulated corneas. Invest Ophthalmol Vis Sci.

[29] Gerber HP (1999). VEGF is required for growth and survival in neonatal mice. Development.

[30] Hayashi S, McMahon AP (2002). Efficient recombination in diverse tissues by a tamoxifen-inducible form of Cre: a tool for temporally regulated gene activation/inactivation in the mouse. Dev Biol.

[31] Carmeliet P (1996). Abnormal blood vessel development and lethality in embryos lacking a single VEGF allele. Nature.

[32] Ferrara N (1996). Heterozygous embryonic lethality induced by targeted inactivation of the VEGF gene. Nature.

[33] Carmeliet P (1999). Impaired myocardial angiogenesis and ischemic cardiomyopathy in mice lacking the vascular endothelial growth factor isoforms VEGF164 and VEGF188. Nat Med.

[34] Stalmans I (2002). Arteriolar and venular patterning in retinas of mice selectively expressing VEGF isoforms. J Clin Invest.

[35] Ruhrberg C (2002). Spatially restricted patterning cues provided by heparin-binding VEGF-A control blood vessel branching morphogenesis. Genes Dev.

[36] Park JE, Keller GA, Ferrara N (1993). The vascular endothelial growth factor (VEGF) isoforms: differential deposition into the subepithelial extracellular matrix and bioactivity of extracellular matrix-bound VEGF. Mol Biol Cell.

[37] Ma IT (2015). VEGF mRNA and protein concentrations in the developing human eye. Pediatr Res.

[38] Feeney SA, Simpson DA, Gardiner TA, Boyle C, Jamison P, Stitt AW (2003). Role of vascular endothelial growth factor and placental growth factors during retinal vascular development and hyaloid regression. Invest Ophthalmol Vis Sci.

[39] Stone J (1995). Development of retinal vasculature is mediated by hypoxia-induced vascular endothelial growth factor (VEGF) expression by neuroglia. J Neurosci.

[40] Penn JS, Madan A, Caldwell RB, Bartoli M, Caldwell RW, Hartnett ME (2008). Vascular endothelial growth factor in eye disease. Prog Retin Eye Res.

[41] Ambati BK (2006). Corneal avascularity is due to soluble VEGF receptor-1. Nature.

[42] Cursiefen C (2004). VEGF-A stimulates lymphangiogenesis and hemangiogenesis in inflammatory neovascularization via macrophage recruitment. J Clin Invest.

[43] Stalmans I (2003). VEGF: a modifier of the del22q11 (DiGeorge) syndrome?. Nat Med.

[44] Schmittgen TD, Livak KJ (2008). Analyzing real-time PCR data by the comparative C(T) method. Nat Protoc.

[45] Fantin A (2010). Tissue macrophages act as cellular chaperones for vascular anastomosis downstream of VEGF-mediated endothelial tip cell induction. Blood.

[46] Pitulescu ME, Schmidt I, Benedito R, Adams RH (2010). Inducible gene targeting in the neonatal vasculature and analysis of retinal angiogenesis in mice. Nat Protoc.

