# Supplementary material for: VEGF188 promotes corneal reinnervation after injury
Source: JCI Insight. 2019 Nov 1;4(21):e130979. doi: 10.1172/jci.insight.130979 (PMC6948769; doi:10.1172/jci.insight.130979)
Supplement: Supplemental data [file jciinsight-4-130979-s128.pdf]

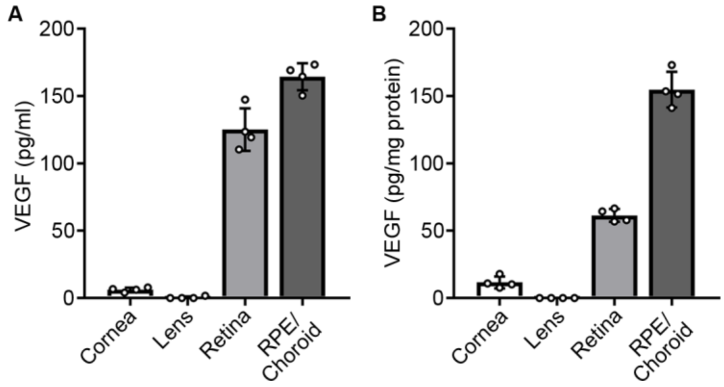

Supplementary Figure 1. VEGF abundance in C57/BL6 ocular tissues.

VEGF protein abundance in the cornea, lens, retina and RPE/choroid of adult C57BL/6 mice, as determined by ELISA, shown (**A**) as mean ± SD per ml of lysate (each tissue was lysed in 150 µl; left) or (**B**) as mean ± SD proportion of total protein in the lysates; n = 4 mice. Each data point represents the value for pooled tissues from both eyes of one mouse.

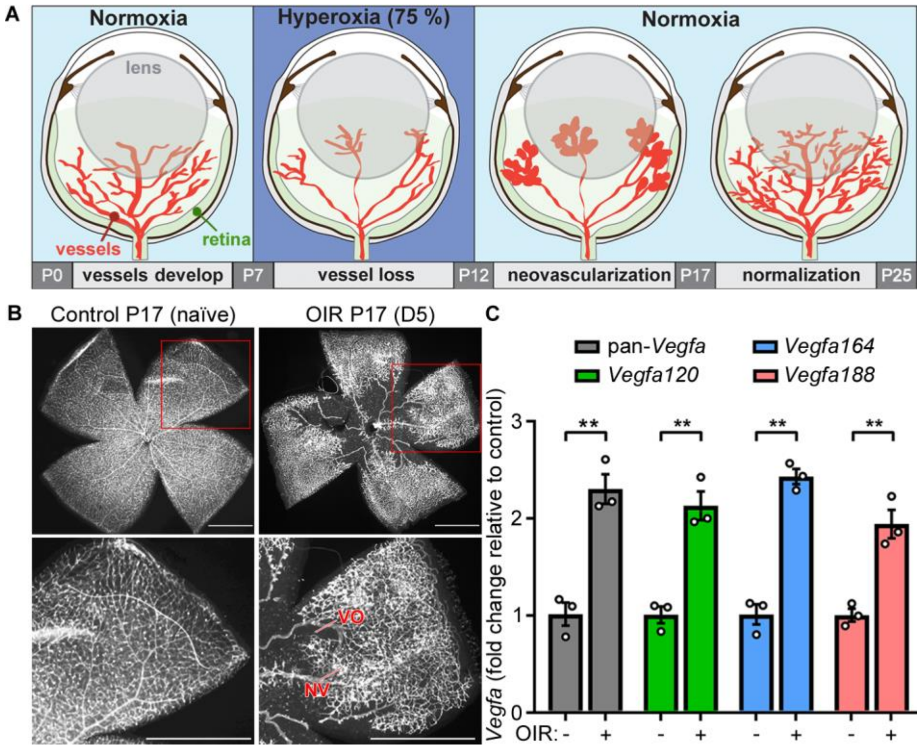

Supplementary Figure 2. *Vegfa* isoform upregulation in the 75% OIR model.

(**A**) Schematic representation of the OIR protocol. Mouse pups were reared in 75% oxygen from P7 - P12 to induce vaso-obliteration in the retina and returned to normoxia on P12. VEGF-driven neovascularization peaks at P17 before onset of vascular normalization.

(**B**) IB4-stained retinal wholemounts from P17 mice housed only in normoxia (control) or subjected to the OIR protocol (D5 after return to normoxia). The red squares indicate the areas shown at higher magnification. Note central vaso-obliteration (VO) and the presence of neovascular tufts (NV) in the OIR model. Scale bars = 1 mm.

(**C**) Total *Vegfa* and *Vegfa* isoform expression in the retinas of P17 mice reared in normoxia (-) or hyperoxia (+). Data are shown as mean fold change ± SD relative to control normoxia; n = 4 mice per age; each data point represents the value for pooled retinas from both eyes of one mouse; one-way ANOVA with Sidak multiple comparison test.

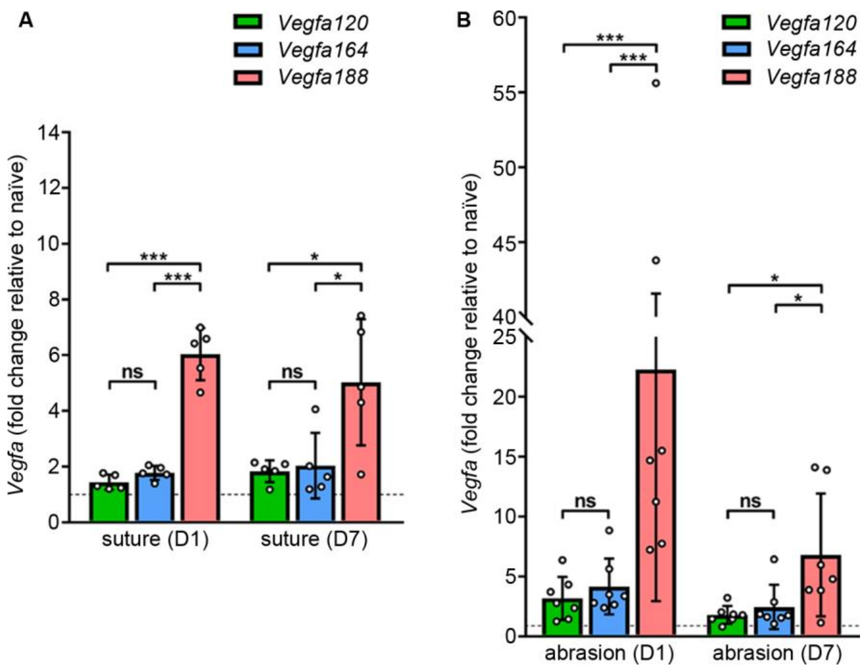

Supplementary Figure 3. *Vegfa* isoform upregulation in models of corneal injury.

(**A,B**) *Vegfa* isoform expression in the corneas of CD1 mice with corneal sutures (**A**) or after corneal abrasion (**B**). Data are shown as mean fold change ± SD relative to *Vegfa* isoform expression in naïve corneas (control); n = 5 mice for each time point in (**A**), n = 7 mice for each time point in (**B**); one-way ANOVA with Tukey multiple comparison test.

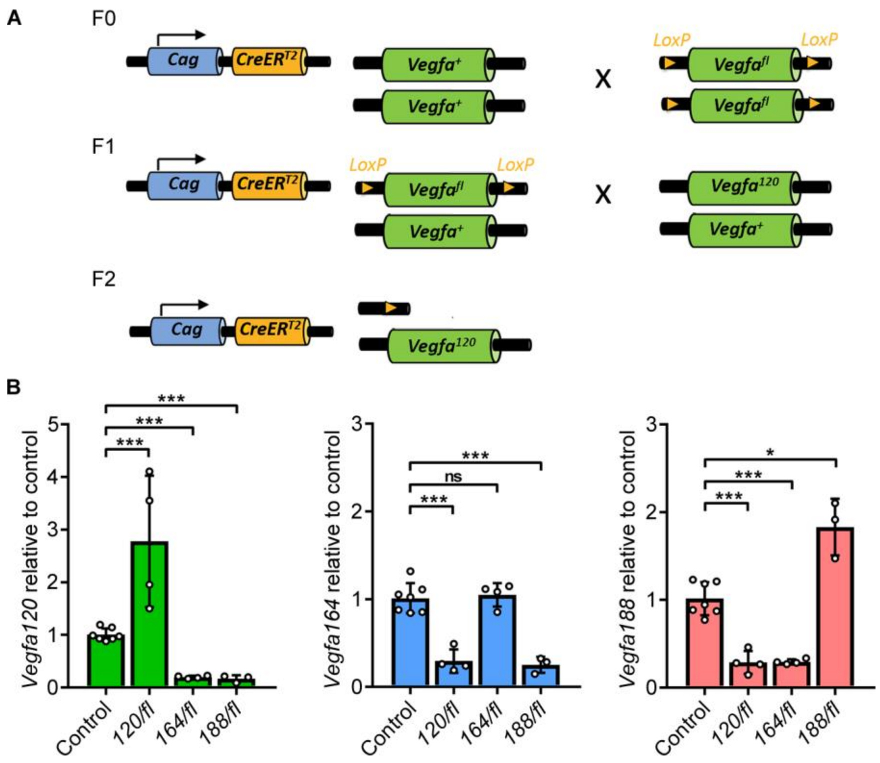

Supplementary Figure 4. Inducible *Vegfa* isoform mutant mice.

(**A**) Mice carrying *Cag-CreER*^{T2} were mated to mice carrying two conditional null (floxed) *Vegfa* alleles. The F1 generation was mated to mice carrying one modified *Vegfa* allele that affects alternative *Vegfa* splicing; only the *Vegfa*^{120} allele is illustrated here. The F2 generation was used for experiments.

(**B**) Validation of *Vegfa* isoform mutants by qRT-PCR of kidney tissue. Data are shown as mean fold change ± SD *Vegfa* isoform expression in *Vegfa*$^{xxx/fl}$*;Cag-CreER*^{T2} (*xxx/fl;Cre+*) relative to controls (as in **B**); n = 4 mice of each genotype; each data point represents the value from one mouse; one-way ANOVA with Dunnet's multiple comparison test.
